# Characterization and expression profiles of WUSCHEL-related homeobox (WOX) gene family in cultivated alfalfa (*Medicago sativa* L.)

**DOI:** 10.1186/s12870-023-04476-5

**Published:** 2023-10-06

**Authors:** Aijiao Xu, Jiaqi Yang, Siqi Wang, Lin Zheng, Jing Wang, Yunwei Zhang, Xiaojing Bi, Hui Wang

**Affiliations:** 1 College of Grassland Science and Technology, China Agricultural University, Beijing, 100193 People’s Republic of China; 2 Beijing Agro-Biotechnology Research Center, Beijing Academy of Agricultural and Forestry Sciences, Beijing, 100097 People’s Republic of China

**Keywords:** Abiotic stress, Expression patterns, *Medicago sativa* L., Phytohormone response, WOX transcriptional factors

## Abstract

The WUSCHEL-related homeobox (WOX) family members are plant-specific transcriptional factors, which function in meristem maintenance, embryogenesis, lateral organ development, as well as abiotic stress tolerance. In this study, 14 MsWOX transcription factors were identified and comprehensively analyzed in the cultivated alfalfa cv. Zhongmu No.1. Overall, 14 putative MsWOX members containing conserved structural regions were clustered into three clades according to phylogenetic analysis. Specific expression patterns of *MsWOXs* in different tissues at different levels indicated that the *MsWOX* genes play various roles in alfalfa. MsWUS, MsWOX3, MsWOX9, and MsWOX13-1 from the three subclades were localized in the nucleus, among which, MsWUS and MsWOX13-1 exhibited strong self-activations in yeast. In addition, various cis-acting elements related to hormone responses, plant growth, and stress responses were identified in the 3.0 kb promoter regions of *MsWOXs*. Expression detection of separated shoots and roots under hormones including auxin, cytokinin, GA, and ABA, as well as drought and cold stresses, showed that *MsWOX* genes respond to different hormones and abiotic stress treatments. Furthermore, transcript abundance of *MsWOX3*, and *MsWOX13-2* were significantly increased after rhizobia inoculation. This study presented comprehensive data on *MsWOX* transcription factors and provided valuable insights into further studies of their roles in developmental processes and abiotic stress responses in alfalfa.

**Supplementary Information:**

The online version contains supplementary material available at 10.1186/s12870-023-04476-5.

## Background

Homeobox (HB) proteins belong to a superfamily of transcriptional factors regulating morphogenesis and development in eukaryotes. The Homeobox domain generally consists of conserved 60–66 amino acids that normally function as a DNA-binding domain, which participates in activating or repressing gene expression [1]. The first HB gene was discovered in *Drosophila melanogaster* and its orthologs were subsequently found in fungi and plants [2, 3]. In plants, the first HB gene was identified from maize, named *KNOTTED-1*, functioning in the division of leaf veins [4]. Plant HB proteins can be classified into 14 groups based on conserved sequence motifs, including BEL, DDT, HD-ZIP I to IV, KNOX, LD, NDX, PLINC, PHD, PINTOX, SAWADEE, and WOX [5].

In higher plants, WOX genes encode WUSECHEL-related homeobox domain, a family of plant-specific transcription factors, which have been already identified and characterized in Arabidopsis, rice, maize, soybean, and other plants [6, 7]. WOX family members were divided into three clades based on phylogenetic evolutionary relationships. The so-called WUS/modern clade exists only in seed plants, the WOX9/intermediate clade exists in vascular plants, and the WOX13/ancient clade was found in vascular and nonvascular plants [8–10].

In the model plant Arabidopsis, fifteen WOX members have been identified and described comprehensively. Compared to the ancient and intermediate clades, the WUS clade contained more members and was investigated more extensively. The first identified *WOX* gene in Arabidopsis was *WUS*, which acts as a conserved vital regulator required for shoot apical meristem maintenance and floral organ development [11]. Orthologs of AtWUS in other species like ROA in Antirrhinum [12], and HDL in Medicago [13, 14] function similarly in maintaining stem cell homeostasis in the shoot apical meristem, however, HDL also regulates leaf blade development in Medicago [14]. WOX1 orthologs including MAW in Petunia [15], STF in Medicago [16], and LAM1 in *Nicotiana sylvestris* [17] play a general role in leaf blade lateral outgrowth and floral organ development by maintaining hormone homeostasis in plants. The WUS clade member WOX2 redundantly acts with the intermediate gene WOX8 in cotyledon boundary and embryo pattern formation [18, 19]. AtWOX3/PRS1 [20] and its orthologs in other plants, MtLFL/MtWOX3 in Medicago [21], OsWOX3A /OsNS in rice [22], NS1 and NS2 in maize [23], and NLD1 in barley [24] are required for the initiation and development of leaves and floral organs, which loss of function led to narrow leaves and petals. Arabidopsis WOX4 and WOX14 promoted gibberellin synthesis and participated in the PXY kinase pathway to regulate procambial stem cell proliferation and xylem differentiation in the vascular tissue [25–27]. WOX5, a paralog of WUS, is specifically expressed in the quiescent center of the root apical meristem and functions similarly to the role of WUS by repressing columellar cell differentiation to control root architecture [28]. PFS2/AtWOX6 is expressed in developing ovules functioning in ovule patterning [29], while rice WOX6 specifies tiller angle by regulating gravitropism and auxin distribution [30]. WOX8, 9, 11, and 12 belong to the intermediate clade. WOX9/STIMPY, in coordination with WOX8, is responsible for maintaining meristematic fate, inflorescence patterning, and embryo expansion [29, 31]. WOX11 and WOX12 are involved in callus formation and root initiation and organogenesis by direct activation of WOX5/7 [32, 33]. The ancient clade contains WOX10, 13, and 14, which play roles in root development, flowering, callus formation, organ reconnection, and drought tolerance [34–37].

Previous reports demonstrated that WOX family members are versatile transcription factors that function in plant growth and development during the whole plant life cycle, from meristem maintenance to embryonic patterning, and from lateral organ formation to abiotic stress tolerance [28, 38–40]. Spatio-temporal expression patterns confer specificities to *WOX* genes during plant growth and development, although they have some common properties [41]. Arabidopsis WUS/Modern clade genes have been demonstrated that they had promiscuous roles to substitute for WUS function in stem cell maintenance in Arabidopsis and for WOX1/STF/LAM1 function in leaf blade expansion [8, 28, 41]. Arabodipsis *WOX5* promoter driving *WUS* to the root meristem or the *WUS* promoter bringing *WOX5* to shoot meristem could complement the *wox5* or *wus* mutant, respectively [28]. Driven by the *MtWOX1/MtSTF* promoter, Arabidopsis WUS clade genes could complement the *lam1* mutant leaf-attenuated phenotypes in *Nicotiana sylvestris* [8]. However, phenotypic recovery of the *wus* mutant by other WUS clade genes in Arabidopsis required accurate expression in the right domain driven by the *WUS* promoter [41]. These observations suggest that the exact expression domains of WOX members are critical for their proper and specific roles.

As sessile organisms, plants are endowed with strong adaptive capacities to adverse environments. Understanding the balance of plant growth and development with abiotic stress tolerance is helpful to improve agricultural productivity. Although WOX transcription factors are well known as development regulators, studies also demonstrated that *WOXs* participate in some abiotic stress responses. Arabidopsis HOS9-1, sharing similarity with WUS and PRS, functions positively in cold stress independent of the well-known CBF pathway [42]. Homologs of WOX13 in cucumber, rice, and Rosaceae, are involved in drought tolerance in like manner [36, 40, 43, 44]. These results indicated that WOX genes play critical roles not only in plant development and growth but also function in abiotic stress responses.

Genome-wide identification and characterization of WOX family genes have been reported in multiple plant species like Arabidopsis, soybean, wheat, sunflower, and a few others [6, 7, 45, 46]. Alfalfa is a high-quality forage legume that is an autotetraploid (2n = 4x = 32) and widely cultivated in the whole worldwide. In this study, we identified and analyzed 14 *MsWOX* genes in the cultivated alfalfa based on a genome-wide scan approach referring to genome data of *Medicago sativa* L. cv. Zhongmu No.1. We predicated their roles by combining analyses of the cis-elements in 3.0 kb promoters and their expression patterns under multiple phytohormonal treatments and abiotic stresses. The study provides a rich resource for further study of MsWOX transcription factors in alfalfa.

## Methods

### Materials, growth conditions, exogenous phytohormones, and stress treatments

Seeds of *Medicago sativa* L. cv. Zhongmu No.1 was germinated in water for 2–5 days, and the subsequent seedlings were planted in a greenhouse at 24 °C (day) and 20 °C (night) under a 16 h light/8 h dark photoperiod, 60–70% relative humidity and a light intensity of 180 µmol·m^− 2^ s^− 1^. Tissues including unfolded leaves, young flowers, mature flowers, nodules, and stems were collected from 12-week-old plants, every sample was harvested from 3 plants at the reproductive stage; and shoots, roots, and shoot apical meristems were harvested from 6 plants at 2-week-old seedlings for each replicate and three biological repeats for RNA extraction.

For hormone treatments, 2-week-old seedlings were transferred to 1/4 Hoagland containing 10 µM 6-BA, 10 µM 2,4-D, 10 µM GA, and 10 µM ABA for 0 h, 6 h, and 12 h, respectively. For abiotic stress treatments, 2-week-old seedlings were shifted into a 4℃ chamber for cold stress for 0 h, 6 h, 12 h, and 24 h; and 2-week-old seedlings were transferred into 10% (w/v) PEG-6000 solution mimicking drought or inoculated by *Sinorhizobium meliloti* 1021 at 0 d,1d,3 d, and 5 d. Shoots and roots of seedlings were split and gathered after the above treatments. 6 plants were collected for each replicate and two biological repeats were performed. All samples were frozen in liquid nitrogen immediately and stored at -80℃ for RNA extraction.

### Identification of alfalfa MsWOXs

MsWOX protein database in alfalfa (cv. ZhongmuNo.1), was downloaded from FigShare https://figshare.com/articles/dataset/Medicago_sativa_genome_and_annotation_files/12623960?file=23754059/ZhongmuNo.1.pros.fasta). Arabidopsis WOX protein sequences were retrieved from Plant Transcription Factor Database (PlantTFDB, http://planttfdb.gao-lab.org/) and used as queries for a local protein blast against ZhongmuNo.1 protein database. In total, 17 sequences were identified with an E-value cut-off of 0.001. After Conserved Domain Search (https://www.ncbi.nlm.nih.gov/Structure/bwrpsb/bwrpsb.cgi) analysis with the 17 proteins and annotating them with Swiss-prot database (https://www.sib.swiss/swiss-prot), 3 proteins were excluded due to lacking proper WUSCHEL-related homeodomain motif.

### Phylogenetic analysis of WOXs

WOX amino acid sequences from rice (Japonica), *Brachypodium distachyon*, and *Medicago truncatula* were downloaded from PlantTFDB (http://planttfdb.gao-lab.org/). Afterward, these downloaded sequences and the identified 14 MsWOXs in alfalfa in this study were aligned using the online tool MAFFT (https://www.ebi.ac.uk/Tools/msa/mafft/). Based on the aligned protein sequences, a neighbor-joining tree was created using MEGA11 with 1000 bootstrap replicates. To make the tree more esthetic, R package ggtree was used.

### Chromosomal location, gene duplication, and synteny analyses of *MsWOX* genes

The chromosomal location information was extracted from the genome annotation file (https://figshare.com/articles/dataset/Medicago_sativa_genome_and_annotation_files/12623960?file=23754059/ZhongmuNo.1.gff) and the visualization was done by TBtools. The duplication events and synteny analyses were performed using MCSanX and displayed with Circos and Dual Synteny Plot in TBtools software. The inter-species synteny analyses were done between alfalfa cv. ZhongmuNo.1 and each of the species; *Brachypodium distachyon*, rice, Arabidopsis, *Medicago truncatula*, and *Glycine max.*

### Gene parameters, conserved motif, and cis-element analyses

The gene structure information including the length of coding sequences and proteins, the number of introns and exons of the 14 MsWOXs, and the corresponding chromosome number were summarized from the GFF annotation file downloaded from ZhongmuNo.1 as described in the chromosomal location. The Molecular Weight (MW) and Isoelectric Point (PI) of the 14 MsWOX proteins were calculated by the ProtParam tool (https://web.expasy.org/protparam/). The length of coding sequences and proteins, the number of introns and exons of the 14 MsWOXs, and the corresponding chromosome number were summarized from the genome annotation file of ZhongmuNo.1 (https://figshare.com/articles/dataset/Medicago_sativa_genome_and_annotation_files/12623960?file=23754059/ZhongmuNo.1.gff). Conserved amino acid motifs of MsWOXs were searched by the online tool MEME Suite 5.1.1 (https://meme-suite.org/meme/), and the motif numbers were set as 10. The phylogenetic tree and conserved motifs of the *MsWOXs* were visualized by Gene Structure View (Advanced) in TBtools [47]. 3.0 Kb upstream genomic sequences before ATG of *MsWOXs* were submitted to the PlantCARE (https://bioinformatics.psb.ugent.be/webtools/plantcare/html/) to predict the putative cis-elements. Simple BioSequence Viewer in TBtools was used to visualize the cis-element distributions on the promoter regions and listed in supplementary Table 1.

### RNA extraction and RT-qPCR

The total RNA of all samples indicated in every experiment was extracted using TRIzol reagent (Invitrogen). The concentration and quality of RNA were tested by NanoDrop (Thermo). 5 µg of total RNA was used as the template for cDNA synthesis with Uni One-Step gDNA Removal and cDNA Synthesis SuperMix (Transgen, AU311) following the manufacturer’s instructions.

The qPCR was performed using a qTOWER3^G^(Analytik Jena) machine with TransStart Green qPCR SuperMix (Transgen, AQ101). The qPCR was carried out in a 96-well optical plate using a Quanstudio Real-time PCR system. Each 10 µl reaction contained 3 µl of diluted cDNA template, 5 µl of 2×SuperMix (Transgen, AQ101), and 2 µl of forward and reverse primers (1 µM). The thermal cycling for amplification was as follows: 5 min at 95 °C, followed by 40 cycles of 95 °C for 10 s and 60 °C for 30 s, and then the melting curve from 60 to 95 °C, 15 s with ΔT 1 °C. Each qPCR reaction was performed in 3 or 4 technical replicates for each biological replicate. The relative gene expression levels were calculated using the 2^−∆∆Ct^ method, and *MsActin* (MsG0380016789.01.T01) was used as an endogenous control for accurate normalization of the qPCR data. Every test was performed with at least two biological repeats. All data were visualized by GraphPad Prism 9.

### Plasmids construction and subcellular localization

The full-length coding sequences (CDS) without stop codon of *MsWUS*, *MsWOX3*, *MsWOX9-1*, and *MsWOX13-1* were amplified using designed primers listed in supplementary Tables 1 and cloned into pMDC83 vector via gateway system (LR, Invitrogen), respectively. The integrity of all recombinant plasmids was confirmed by sequencing. Then the successful recombinant plasmids of *MsWOXs-GFP* were introduced into agrobacteria GV2260 using freeze-thaw transformation and transiently expressed in *N. benthamiana* leaves mediated by infiltration of GV2260. Subcellular localization was observed after infiltrating 48 h by confocal microscopy (Nikon, TE2000-E).

### Transcriptional activation test in yeast

The full-length CDS of *MsWUS*, *MsWOX3*, *MsWOX9*, and *MsWOX13-1* were amplified and cloned into pGBKT7-BD vector via a gateway system (LR, Invitrogen) as baits. Each bait clone with the empty prey vector pGADT7 was co-transformed into the yeast strain Gold using PEG methods. pGBKT7-p53 co-transformed with pGADT7 was used as a positive control and pGBKT7-lam with pGADT7 as a negative control. All these co-transformed cells were diluted to a gradient concentration of 10^− 0^, 10^− 1^, 10^− 2^, and 10^− 3^, and then dropped on the SD/-Trp/-Leu/-His + X-a-gal solid medium for incubating at 30 °C for 3 days.

### Statistical analysis

Error bars in qPCR show the SD of three or four biological or technical repeats, as indicated in the legends. Experimental data were subjected to one-way analysis of variance (ANOVA) and post hoc LSD tests to determine significant differences among mean values at the probability level of 0.05.

## Results

### Identification and characterization of *MsWOX* genes in alfalfa

To isolate MsWOX members in alfalfa, 15 Arabidopsis AtWOX protein sequences were retrieved to perform a protein blast against the alfalfa protein database. In total, 17 proteins were identified in cultivated alfalfa cv. Zhongmu No.1. The conserved domain search and a protein blast with the protein database SwissProt verified the characteristics of these 17 members. Besides the Homeobox or Homeobox Superfamily domain, three of the 17 genes, including MsG0280011271, MsG0280007695, and MsG0380017666, contained a bZIP domain, which suggested that they belong to another family named HD-ZIP Family. Therefore, we excluded them from the WOX family, and 14 MsWOX proteins were finally identified in alfalfa cv. Zhongmu No.1.

### Phylogenetic analysis of the MsWOX members

**Fig. 1:**
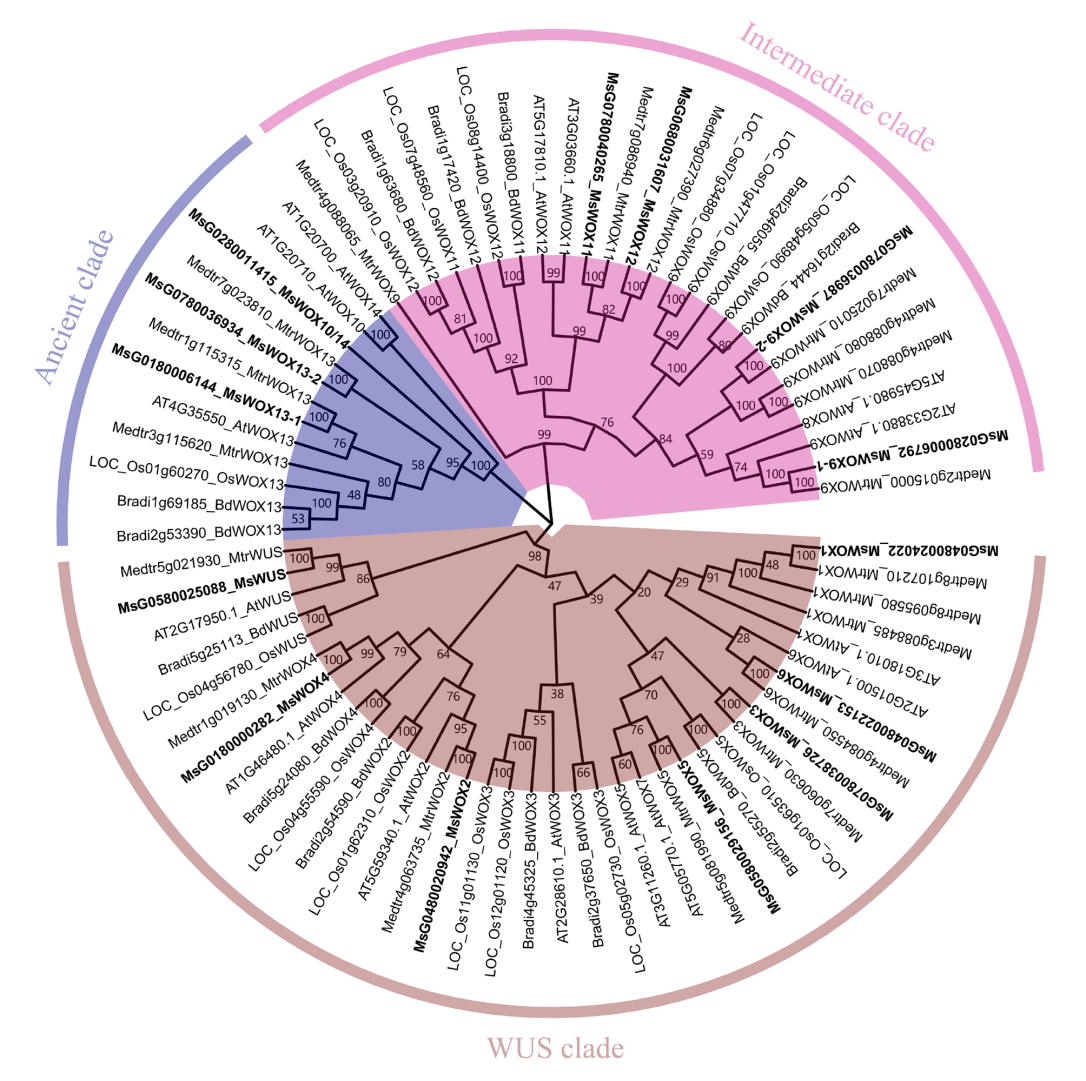
The phylogenetic unrooted tree of WOXs. Full length of WOX proteins from *A*. *thaliana* (At), rice (Os), *B*. *distachyon* (Bd), *M. truncatula* (Mt), and *M. sativa* (Ms) were aligned using MAFFT, and the phylogenetic tree was constructed using MEGA11 with 1000 bootstrap replicates. The ancient clade, the intermediate clade, and the WUS clade were highlighted by purple, pink, and brown sectors

To identify the evolutionary relationships of the WOXs, the 14 alfalfa MsWOX proteins (cv. ZhongmuNo.1) together with 15 AtWOXs in Arabidopsis, 19 MtWOXs from *Medicago truncatula*, 13 BdWOXs in *Brachypodium distachyon* and 14 OsWOX in rice (cv. Japonica), were aligned to construct a phylogenetic tree based on neighbor-joining method with 1000 replicates. These 75 WOX proteins were classified into 3 clades (Fig. 1), i.e., the ancient clade, the intermediate clade, and the WUS clade, which is consistent with the classification of subclades in Arabidopsis [9].

Due to the comprehensive functional understanding of AtWOXs, we named the 14 putative MsWOXs according to the closest Arabidopsis or *Medicago truncutula* WOX homologs (Fig. 1). The ancient clade had the lowest number of MsWOXs with three members, including MsG0280011415_MsWOX10/14, MsG0180006144_MsWOX13-1, MsG0780036934_MsWOX13-2. The intermediate clade contained four MsWOX members, including MsG0280006792_MsWOX9-1, MsG0780036987_MsWOX9-2, MsG0780040265_MsWOX11, and MsG0680031607_MsWOX12. The largest number of MsWOXs were grouped into the WUS/Modern clade, containing the half number of the total MsWOXs, including MsG0580025088_MsWUS, MsG0480024022_MsWOX1, MsG0480020942_MsWOX2, MsG0780038726_MsWOX3, MsG0180000282_MsWOX4, MsG0580029156_MsWOX5, and MsG0480022153_MsWOX6 (Fig. 1). Next, we analyzed the properties of *MsWOX* genes, which harbor various numbers of exons and introns, encoding proteins that range in size from 21.2 KDa to 94.4 KDa, with isoelectric points ranging from 5.02 to 9.79 (Table 1).

**Table 1:** Characteristics of *MsWOX* genes in *Medicago sativa* L. cv. Zhongmu No.1

Gene	Gene ID	Chr	animo acid (aa)	CDS (bp)	No. of introns/exons	MW (Da)	pI
MsWUS	MsG0580025088.01	5	306	918	2/3	34532.93	6.41
MsWOX1	MsG0480024022.01	4	376	1128	3/4	43056.89	8.42
MsWOX2	MsG0480020942.01	4	233	699	2/3	25965.02	5.58
MsWOX3	MsG0780038726.01	7	203	609	1/2	23472.36	9.32
MsWOX4	MsG0180000282.01	1	187	561	3/4	21561.34	9.79
MsWOX5	MsG0580029156.01	5	184	552	1/2	21209.71	6.53
MsWOX6	MsG0480022153.01	4	409	1227	3/4	47097.22	8.52
MsWOX9-1	MsG0280006792.01	2	315	945	4/5	34498.83	6.98
MsWOX9-2	MsG0780036987.01	7	417	1251	2/3	46428.49	5.53
MsWOX11	MsG0780040265.01	7	296	888	2/3	32286.49	7.67
MsWOX12	MsG0680031607.01	6	262	786	1/2	27602.35	8.81
MsWOX13-1	MsG0180006144.01	1	274	822	1/2	31285.17	6.43
MsWOX13-2	MsG0780036934.01	7	418	1254	4/5	47489.29	9.37
MsWOX10/14	MsG0280011415.01	2	832	2496	9/10	94413.62	5.02

### Conserved motifs analysis

The Conserved Domain Search and MEME tools were used to dissect the gene structures and the conserved domains and motifs of the 14 MsWOXs. All MsWOXs contained the Homeobox or HD Superfamily domain, which is distributed at different positions of those proteins, contributing to the common features of the WOX family in alfalfa (Figure S1). In addition to the large HD, other smaller conserved but specific motifs are also apparent. Members of the WUS clade contained conserved domains at the C-terminal designated as motif 6. The intermediate MsWOXs owned a clade-specific motif 5 at the C-terminal, while the ancient clade contained a peculiar N-terminal motif 4 (Figure S1). The specific motifs might contribute to the specialized biological functions of members in the corresponding clade.

### Chromosomal localization and synteny analyses

The *MsWOX* genes were unevenly distributed on 6 chromosomes of alfalfa cv. ZhongmuNo.1. Four genes were located on Chr7, followed by three genes on Chr4, two genes on Chr 1, Chr2, and Chr5, and one gene on Chr6. *MsWOXs* were not identified on Chr3 and Chr8 based on our analysis (Figure S2).

Gene duplication events drive the evolution of species by increasing the number of functional genes [48]. We conducted intra-species and inter-species genome synteny analyses. Among the 14 MsWOXs, only two pairs, pair of MsWOX12 and MsWOX11 and pair of MsWOX2 and MsWOX3, showed intra-genomic collinearity, suggesting segmental duplication occurred in the alfalfa cv. ZhongmuNo.1 genome (Figure S3A). By the inter-species synteny analysis, 9, 14, 14, and 3 MsWOXs were syntenic with those of Arabidopsis, *Medicago truncatula*, *Glycine max*, and *Oryza sativa*, respectively. The result suggests that there might be genome duplication in *Glycine max* compared to alfalfa cv. Zhongmu No.1 because at least two orthologs of each MsWOX were found in *Glycine max* (Figure S3B).

### Tissue-expression patterns of *MsWOXs* in alfalfa

**Fig. 2:**
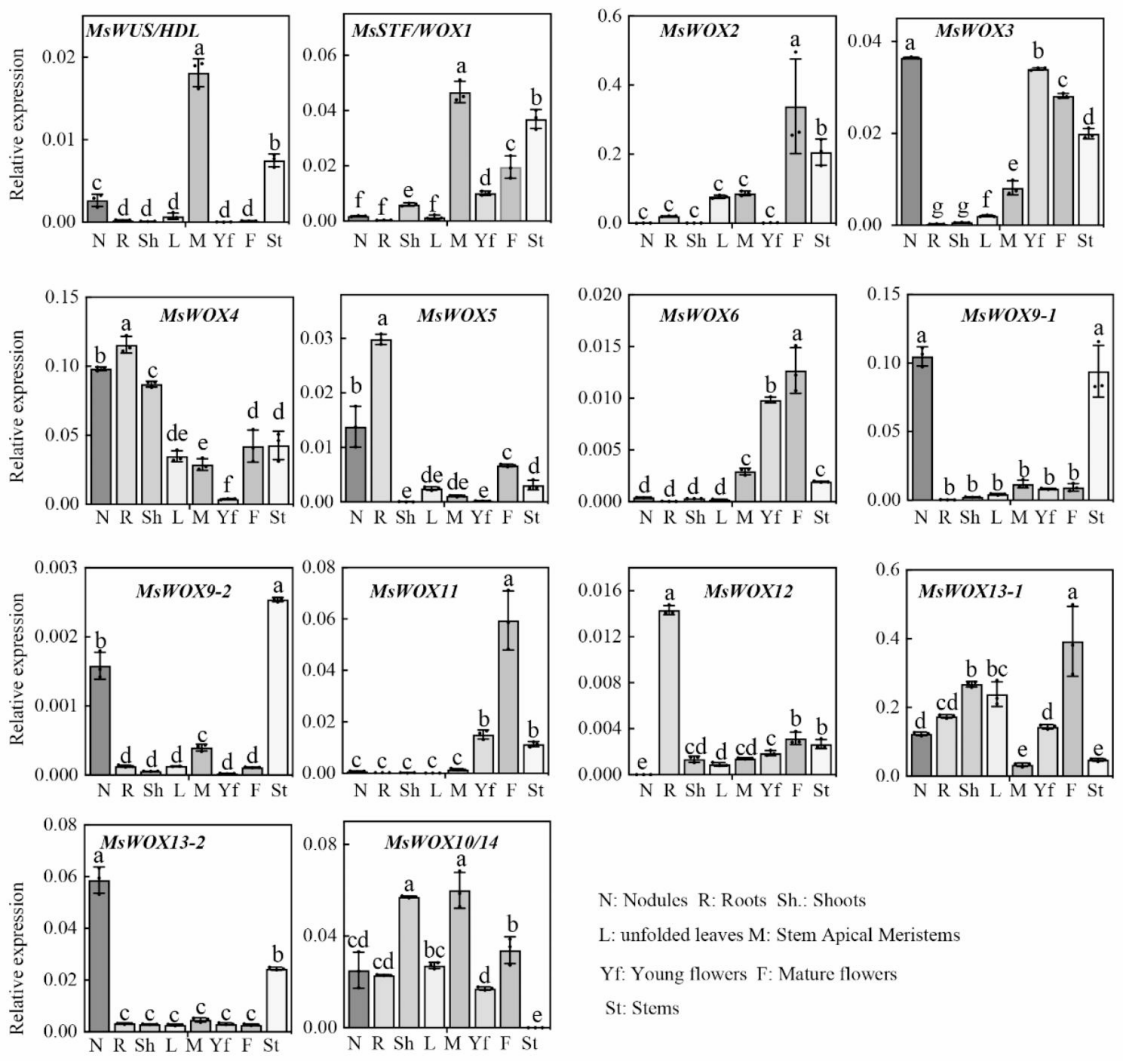
Tissue-specific expression patterns of 14 *MsWOXs*. The relative expression levels of *MsWOXs* in different tissues were detected by RT-qPCR. N, Nodules; R, 10-day-old seedling roots; Sh, 10-day-old seedling shoots; L, Unfolded leaves; S, Stem apical meristems; Yf, Young flowers; F, Mature flower; St, Stems. R, Sh, and M were collected from 3 individuals of 2-week-old plants at the vegetative stage, while other tissues were harvested from six independent 12-week-old plants at the reproductive stage. *MsActin* was used as an internal control. Values are means of three biological repeats ± SD, and two biological experiments repeated

The specialized function of individual *MsWOXs* is probably indicated by their spatial expression profiles [8, 28, 41]. To dissect the expression patterns of alfalfa *MsWOX* genes, eight different tissues, i.e., shoots, shoot apices, and roots of 2-week-old seedlings, unfolded leaves, young flowers, mature flowers, nodules, and stems of 12-week-old plants were collected and tested by RT-qPCR. The results showed that different expression patterns of *MsWOX* genes were displayed in different organs at different stages (Fig. 2). *MsWUS* and *MsWOX1*, two WUS/modern clade genes, were highly and specifically accumulated in the shoot apex, which is consistent with WUS conserved roles in meristem maintenance and that of *WOX1* in young leaf expansion. Transcripts of *MsWOX2*, *MsWOX3, MsWOX6, MsWOX11*, and *MsWOX13-1* displayed higher levels in mature flower organs, while *MsWOX2, MsWOX4*, and *MsWOX5* showed lower expression in young flowers, suggesting that these genes are required at different stages of reproductive organs development. Intermediate clade genes *MsWOX9-1* and *MsWOX9-2*, and the ancient clade gene *MsWOX13-2 exhibited* similar patterns in being especially expressed in nodules and stems. On the other hand, *MsWOX12* was strongly expressed only in roots. *MsWOX4* and *MsWOX5* also displayed higher abundance in roots (Fig. 2). Furthermore, *MsWOX4* and two ancient genes of *MsWOX13-1* and *MsWOX10/14* showed considerable transcript accumulation in almost all organs except *MsWOX4* in young flowers and *MsWOX10/14* in stems (Fig. 2).

### Subcellular location and self-activation activities of MsWOXs

**Fig. 3:**
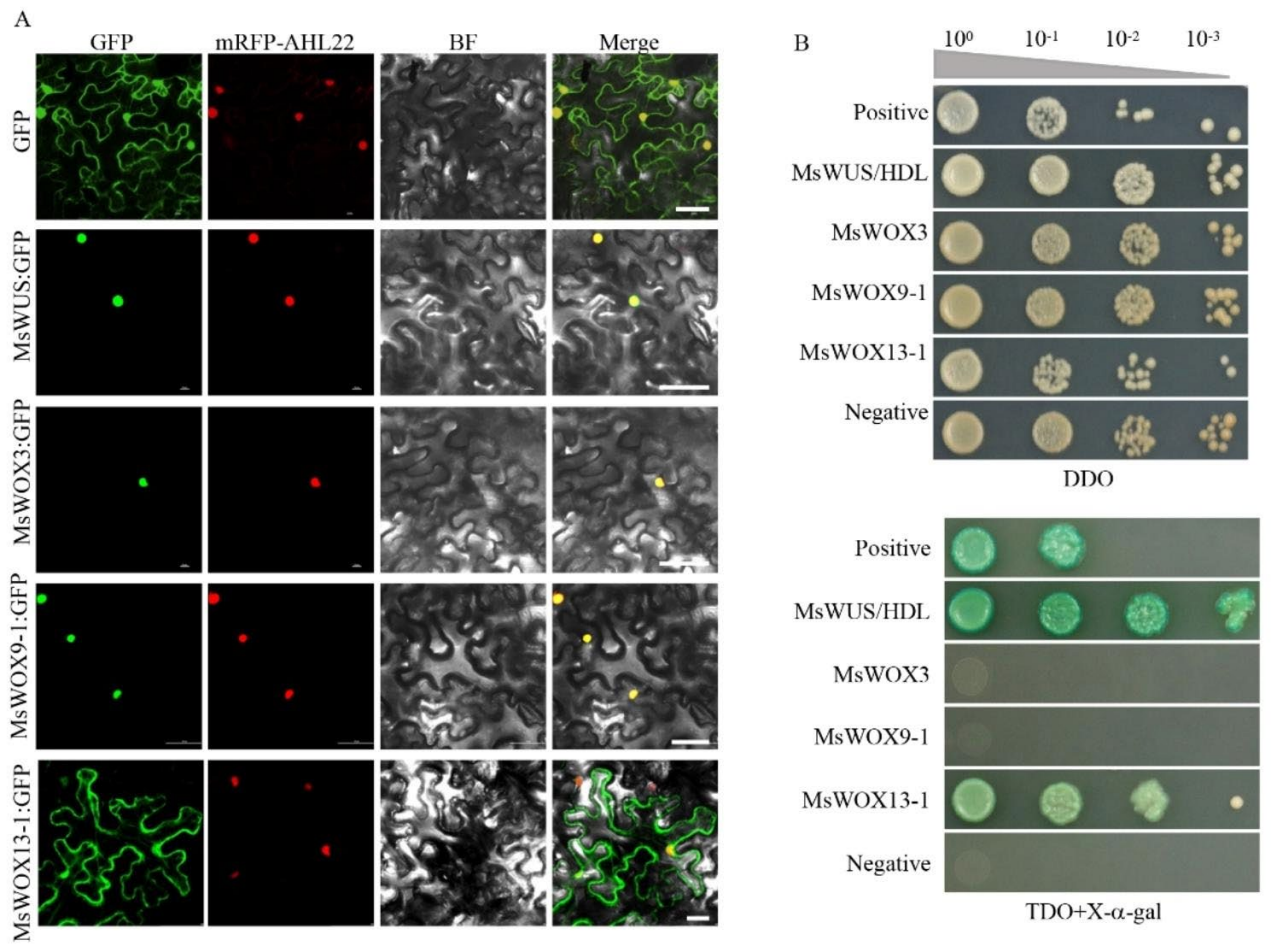
Subcellular localization and transactivation assay of MsWOXs from different clades. **A**: Subcellular location of MsWOXs in tobacco epidermal cells. Recombinant plasmids of GFP, MsWUS-GFP, MsWOX3-GFP, MsWOX9-1-GFP, and MsWOX13-1-GFP driven by 35S promoter were transiently expressed in *N.benthamiana* epidermal cells for 48 h and fluorescent signals were detected by confocal microscope. GFP: Green fluorescent signal; mRFP-AHL22: nuclei-localized marker RFP; BF: bright field; Merge: merged microscope of GFP and RFP. Bars = 50 μm. **B**: Transcriptional activity of MsWOXs in yeast cells. Full-length CDS of *MsWOXs* were fused with GAL4 DNA-binding domain (BD) as baits and then transformed to yeast strain with prey. The co-transformed cells were diluted to 10^0^,10^–1^,10^− 2^,10^–3^ indicated in a gray triangle, and drops were deposited on SD/-Trp-Leu-His + X-a-Gal. Positive control: pGBKT7-p53, negative control: pGBKT7-lam.

MsWOXs are members of the already established WOX family of transcription factors, although no clear nuclear localization signals (NLS) were predicted using PSORT and PredictNLS. To gain better insights into the biological roles of MsWOX proteins, we investigated the subcellular localization and self-activation of four MsWOXs selected from the three clades. The full-length coding sequences of MsWUS, MsWOX3, 9 − 1, and 13 − 1 were cloned individually and fused to GFP-containing vectors, which were then infiltrated into *Nicotiana benthamiana* leaves, and fluorescence signals were examined in leaf epidermal cells. We detected that MsWUS, MsWOX3, and MsWOX9-1 were nuclear-localized, while MsWOX13-1 was targeted to the nucleus and cytoplasm similar to the 35 S: GFP control (Fig. 3A), indicating that MsWOXs possess the basic nuclear-localization property as transcription factors (TF). In addition to being nuclear-targeted, self-activation is another characteristic of TFs. To further explore this, full CDS of *MsWUS*, *MsWOX3*, *MsWOX9-1*, and *MsWOX13-1* were cloned in the pGBKT7 plasmid as baits, and p53-pGBKT7 was used as the positive control. All the above-regenerated plasmids with empty pGADT7 were co-transformed into the Y2HGold yeast strain using the PEG method. All combinations of MsWOXs and pGADT7 could grow in SD/-Trp-Leu medium, but only the positive control, MsWUS, and MsWOX13-1 could grow and display strong staining activities on SD/-Trp-Leu-His + X-a-gal (Fig. 3B), indicating that MsWUS and MsWOX13-1 have self-activation activities while MsWOX3 and MsWOX9-1 do not, at least in yeast test system.

### Cis-elements recognition in the promoters of *MsWOXs*

**Fig. 4:**
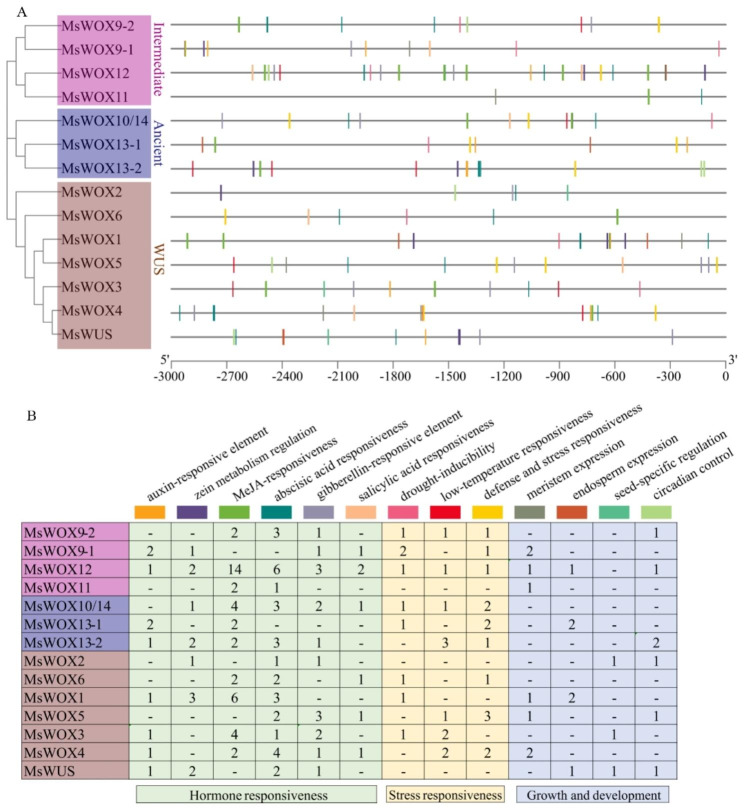
Cis-acting elements of *MsWOX* promoters. **A**: The cis-acting elements architectures in the 3.0 Kb *MsWOX* promoters. **B**: The numbers of cis-elements in the *MsWOX* promoters. Rectangles with different colors represented different cis-acting elements. Light green, yellow, and purple orthogons indicated hormone responsiveness, stress responsiveness, and growth and development-related elements

Analyses of the cis-acting elements on promoters could provide information on regulators of gene transcriptional levels, which is essential to improve our understanding of gene regulations to reveal their biological functions. We analyzed 3.0 kb putative promoter regions upstream of the translation start site of each of the 14 *MsWOX* genes in alfalfa. Numerous cis-acting elements related to the control of different biological processes were observed in all promoters, including plant growth and development conferring meristem and endosperm expression, phytohormone-responsive regulators such as MeJA, salicylic acid, auxin, gibberellin, and ABA, as well as abiotic stress-responsive motifs involving low-temperature, drought, and wound response (Fig. 4A, B). In total, we identified 24 cis-acting elements for growth and development involving meristem, endosperm, seed, and circadian regulation. Besides, 40 jasmonic acid (JA) and 7 salicylic acid-responsive elements existed in the promoters, implying the possible involvement of *MsWOXs* in biotic stresses. Drought-inducibility and low-temperature responsive elements were observed in 8 and 7 *MsWOX* promoters, respectively. In addition, GA, auxin, and ABA-responsive elements were identified in 10, 8, and 12 *MsWOX* promoters, respectively, (Fig. 4B), suggesting that the expression of most of the *MsWOX* genes may be controlled by phytohormones in response to developmental, biotic, or abiotic signals.

### Expression of alfalfa *MsWOX* genes respond to different exogenous phytohormones

**Fig. 5:**
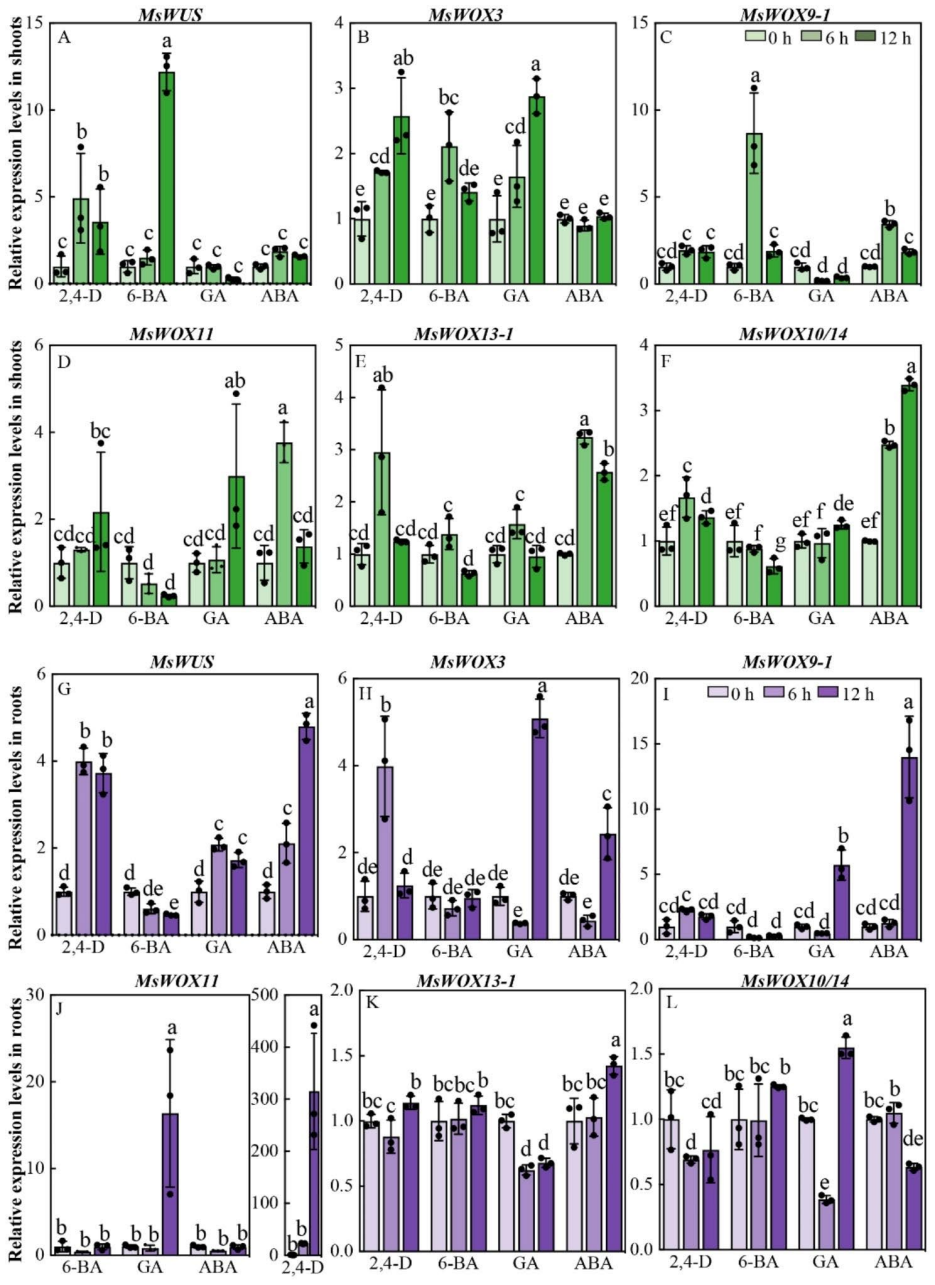
Relative expression levels of the *MsWOXs* in response to different exogenous hormone treatments. Transcripts alteration of *MsWOXs* from three subclades under different hormone treatments. 2-week-old seedlings were transferred to Hoagland solutions containing 10 µM 2,4-D, 6-BA, GA, and ABA, respectively. The shoots (green bars) and roots (purple bars) were collected separately at 0 h, 6 h, and 12 h after the indicated treatments. The relative expression levels were tested by RT-qPCR and calculated from three repeats relative to the non-treatment (0 h). Values are means of three technical repetitions ± SD and two biological replicates performed

*WOX* genes have been well described for their close affiliation to phytohormones in plant growth and development [49]. Here we also found multiple hormone-responsive elements distributed in various *MsWOX* promoters, prompting us to investigate the relationships between *MsWOX* gene expression and phytohormones. To explore how *MsWOX* genes respond to different hormones, 2-week-old seedlings grown in 1/4 Hoagland solution were transferred to different exogenous phytohormone treatment conditions (10 µM 2,4-D, 6-BA, GA, and ABA each), and shoots and roots were split and harvested for detecting transcriptional alteration of *MsWOXs*. Six *MsWOXs* from three clades (*MsWUS* and *MsWOX3* from the WUS clade, *MsWOX9-1* and *MsWOX11* from the intermediate clade, and *MsWOX13-1* and *MsWOX10/14* from the ancient clade) were selected for subsequent analysis by RT-qPCR. Expression of *MsWUS*, *MsWOX3*, *MsWOX13-1*, and *MsWOX10/14* were 2,4-D induced slightly in shoots (Fig. 5A, B, E, F), while transcripts of *MsWUS*, *MsWOX3*, and *MsWOX11* were upregulated obviously in roots under exogenous 2,4-D treatment (Fig. 5G, H, J). Cytokinin 6-BA positively affected expression levels of *MsWUS*, *MsWOX3*, and *MsWOX9-1* but negatively regulated *MsWOX11*, *MsWOX13-1*, and *MsWOX10-14* in shoots (Fig. 5A-F), as well as reduce the accumulation of *MsWUS* and *MsWOX9-1* in roots at mild degree (Fig. 5G, I). Interestingly, the ancient clade genes of *MsWOX13-1* and *MsWOX10/14* were insensitive to 6-BA in roots (Fig. 5K, L). In addition, all *MsWOX* transcripts in roots that we detected were upregulated by GA except for *MsWOX13-1* which was reduced (Fig. 5G-L), however, *MsWOXs* were insensitive to GA in shoots except for *MsWOX3* and *MsWOX11* (Fig. 5A-F). Besides, we found that ABA activated the expression levels of *MsWOX9-1* and *MsWOX13-1* in both shoots and roots (Fig. 5C, E, I, K), but only induced respective transcripts of *MsWOX11* in shoots and *MsWOX3* in roots shown in Fig. 5D H. Additionally, the expression of *MsWOX10/14* was significantly accumulated in shoots but reduced in roots by ABA (Fig. 5F, L). These results indicate that *MsWOX* genes are responsive to different phytohormone treatments with distinct expression patterns.

### Responses of *MsWOX* genes to abiotic stresses

**Fig. 6:**
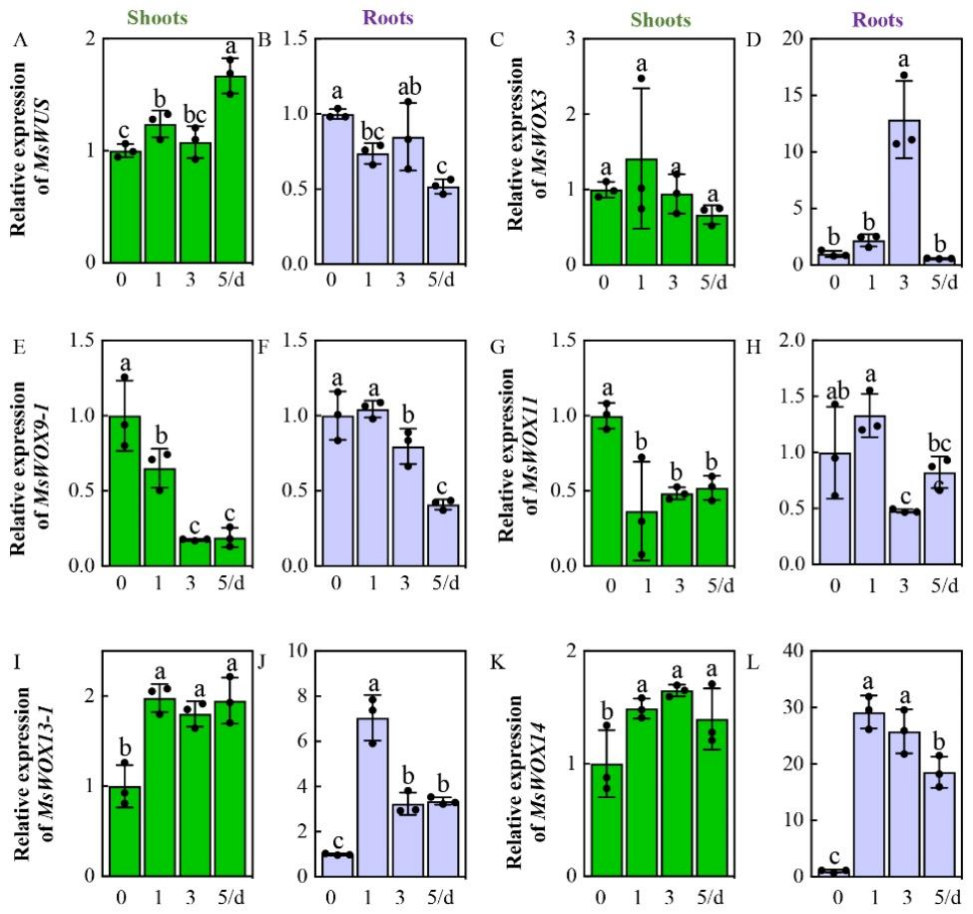
Transcripts of*MsWOXs* in response to mimicking drought stress. Expression levels of *MsWOXs* under PEG-6000 treatment mimicking the drought stress. 2-week-old seedlings were transferred to Hoagland solution containing PEG-6000 (10%, w/v). The shoots (green bars) and roots (purple bars) were collected separately at 0 d, 1 d, 3 d, and 5 d after the PEG treatment. The relative expression levels were tested by RT-qPCR and calculated from three repeats relative to the non-treatment (0 d). Values are means of three technical repetitions ± SD and two biological replicates performed

Because we identified 9 motifs responsive to drought and 11 motifs responsive to low temperature in the in-silico analysis of cis-acting elements in *MsWOXs* promoters, we investigated the dynamic expression patterns of *MsWOXs* under PEG 6000 and 4 ℃ mimicking drought and low-temperature conditions, respectively. Firstly, 2-week-old alfalfa seedlings were transferred to 10% PEG 6000 in 1/4 Hoagland solution to mimic drought. After PEG treatment, expression of *MsWUS* was slightly induced in shoots but suppressed in roots, in which both induction in shoots and repression in roots peaked at 5 days after PEG-6000 treatment (Fig. 6A, B). Another WUS clade gene *MsWOX3* positively responded to PEG-6000 in roots with the highest accumulation on the third day, while no significant changes were detected in shoots (Fig. 6C, D). Interestingly, two intermediate clade members *MsWOX9-1* and *MsWOX11* were reduced in both shoots and roots (Fig. 6E-H). The ancient clade member *MsWOX13-1* transcripts were activated by PEG-6000 in both shoots and roots (Fig. 6I, J), while the other ancient clade member *MsWOX10/14* was visibly upregulated in roots under PEG treatment but slightly altered in shoots (Fig. 6K, L).

**Fig. 7:**
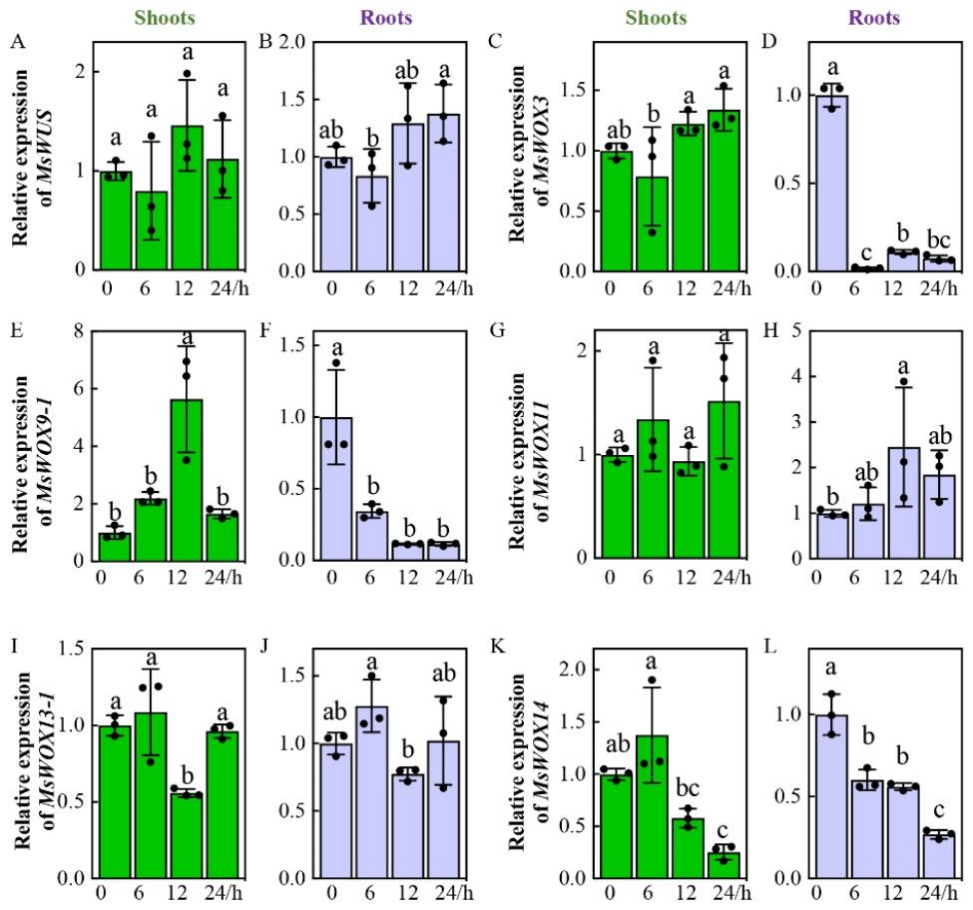
Relative expression levels of six *MsWOX*genes in cold-treated seedings. Transcripts of *MsWOXs* in response to chilling. 2-week-old seedlings were transferred to a low temperature (4℃) chamber for another 0 h, 6 h, 12 h, and 24 h. The shoots (green bars) and roots (purple bars) were collected separately after 4℃ treatments. The relative expression levels were tested by RT-qPCR and calculated from three repeats relative to the non-treatment (0 d). Values are means of three technical repetitions ± SD and two biological replicates performed

Next, we tested how alfalfa *MsWOXs* respond to chilling stress because 11 low-temperature responsive cis-elements were distributed to seven *MsWOX* promoters including *MsWOX3*, *9 − 1*, and *10*/*14* (Fig. 4A, B). 2-week-old seedlings were shifted to 4℃ incubators for 6, 12, and 24 h for transcripts detection. We found that *MsWUS* and *MsWOX11* failed to respond to low temperatures in both shoots or roots (Fig. 7A, B, G, H). Expression of *MsWOX3*, *9 − 1*, and 10/*14* in roots were all downregulated under 4 ℃ treatment (Fig. 7D, F, L), especially the root transcripts of *MsWOX3* were reduced dramatically (Fig. 7D). *MsWOX9-1* expression in shoots was enhanced after 4℃ treatments at 6 and 12 h and then recovered to the level of pre-treatment at 24 h (Fig. 7E), and two ancient genes *MsWOX13-1* and *MsWOX10/14* were decreased clearly in shoots compared to the control (Fig. 7I, K).

### Transcriptional response of *MsWOX* genes to rhizobium inoculation

**Fig. 8:**
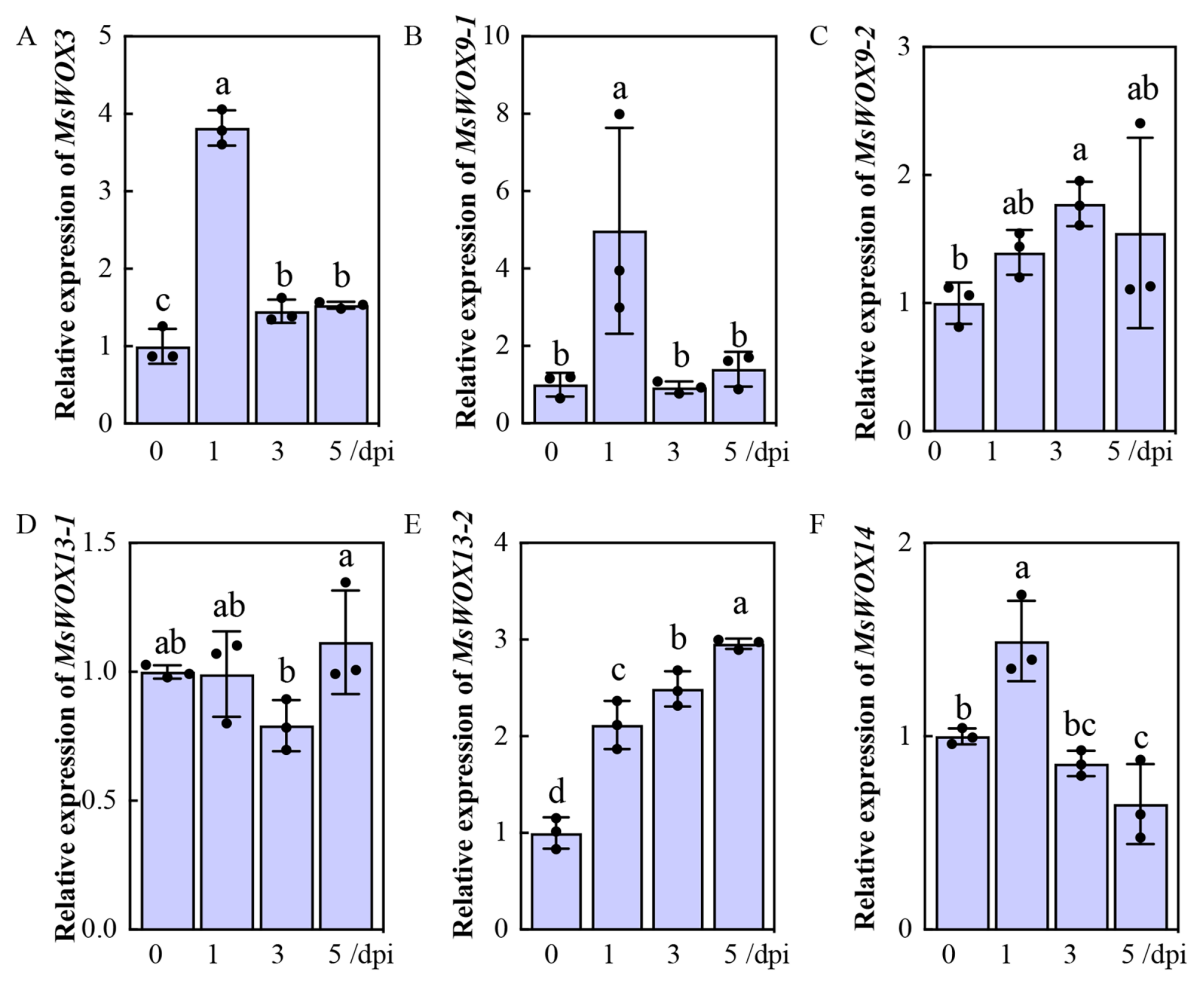
Relative expression levels of six *MsWOX * genes in rhizobium inoculated seeding roots. 10-day-old seedlings inoculated by rhizobium (*Sinorhizobium meliloti 1021*, OD_600_ = 0.01) under low-nitrogen Hoagland solution. The underground part of the seedlings was sampled at day 0, 1, 3, and 5 dpi (days post inoculation) for expression level detection of *MsWOXs*. Values are means of three technical repetitions ± SD and two biological replicates performed

Under nitrogen deficiencies in soil, legumes could convert atmospheric nitrogen into ammonium through symbioses with rhizobia, and this nodulation is initiated by the infection of root hairs by rhizobia forming the nodule primordia from root cortices. To gain insight into the roles of *MsWOXs* in nodulation, 2-week-old seedlings were inoculated with rhizobia *Sinorhizobium meliloti* 1021, and the infected roots were harvested from 1-, 3-, or 5-days post inoculation (dpi) for further RT-qPCR analysis. Six *MsWOXs* which were highly accumulated in nodules were analyzed (Figs. 2 and 8). Among them, the relative expression levels of *MsWOX3*, *9 − 1*, and *10/14* were upregulated transiently at 1dpi (Fig. 8A, B, F), while transcripts of *MsWOX13-2* were steadily accumulated from 1 to 5 dpi (Fig. 8E). However, *MsWOX13-1* levels showed no obvious changes in response to rhizobium compared to uninoculated roots (Fig. 8D). These results suggest that *MsWOXs* may be involved in nodulation. Taken together, our results uncover the type and behavior of WOX genes in the economically important crop alfalfa and provide functional insights into plant development, hormonal signaling, abiotic stress response, and symbiotic nitrogen fixation.

## Discussion

### Alfalfa WOX family members have highly conserved functions

The number of WOX family members varies from species to species, but they are conserved and fall into three distinct subclades via phylogenetic analysis [7, 9]. The WOX family transcription factors play essential roles in plant growth and development, from stem cell maintenance at meristem (WUS in shoot apical meristem, WOX4 in procambial meristem, WOX5 in root apical meristem) to embryo patterning [11, 28], from development of lateral organs to somatic embryogenesis [8, 34]. Since the discovery of Arabidopsis WUS, several *WOX* genes have been characterized and studied extensively in different species, including wheat, cotton, cucumber, *Brachypodium*, etc. [50–53]. In this study, AtWOX protein sequences were used as a query for BLAST search, and 14 genes harboring WOX homeodomains were identified, which were grouped into three subclades (Fig. 1). This classification is consistent with other reported plants. Even though a previous study identified 34 *MsWOXs* genes in alfalfa using homeodomain sequences as a query and named them according to their location on the chromosomes [54], here we identified 14 MsWOXs using comprehensive bioinformatic analysis and named them based on homology to Arabidopsis and Medicago WOX genes (Fig. 1), which is helpful to explore novel and established biological functions. Nuclear localization is a prerequisite for a protein to act as a transcription factor since eukaryotic transcription occurs in the nucleus. Furthermore, the presence or absence of self-activation property is very important information in determining protein-protein interaction as most transcription factors interact with other proteins to perform their functions. Our localization studies using GFP fusions revealed that the GFP signals of MsWUS, MsWOX3, and MsWOX9-1 were targeted to the nucleus, while MsWOX13-1 was localized in the nucleus and cytoplasm (Fig. 3A), which might be caused by the specialized ancient motif 4 in MsWOX13-1 compared to other subclades (Figure S1). From yeast self-activation test results, we found that MsWOX3 and MsWOX9-1 did not display activities, which is consistent with previously reported MtWOX3/MtLFL interacting with TPL as a transcriptional repressor [21]. The loss of motif 3 in the intermediate member of MsWOX9-1 might be the reason for its lack of self-activation (Figure S1), which could be valuable to investigate in further study.

### Expression patterns of MsWOXs

Although WOX members contain a conserved homeodomain, they carry out a variety of roles in plant development. WUS clade genes can substitute for WUS and WOX1 functions in shoot meristem maintenance and leaf blade expansion, respectively, but the native promoters of *WUS* and *WOX1* are required for complementing the respective mutants [8, 41], indicating that specific expression profiles are the key factors for the specific functions of WUS clade members. Tissue-specific expression profile analysis uncovered that *MsWUS* conservatively expressed in the shoot apical meristem, and *MsWOX5* displayed conserved expression patterns in the root apical meristem (Fig. 2), suggesting the respective conserved roles in controlling shoot and root meristem maintenance. Previous research reported that orthologs of WOX3 analogously function in leaf development in maize, rice, and barley [24, 55], whereas the loss-of-function *mtwox3/lfl* mutant in *Medicago truncatula* conferred the loose-flower phenotypes [21]. *MsWOX3* also showed higher expression in alfalfa flowers (Fig. 2), indicating that the role of *WOX3* may be restricted to leaf blade development in monocots, but in eudicots at least in Arabidopsis, Medicago, and alfalfa *WOX3* function could be involved in floral organ development. In addition, *MsWOX3* was highly expressed in nodules (Fig. 2) and induced after inoculation of rhizobia (Fig. 8A), suggesting that *MsWOX3* function is diversified in flowers and nodules in alfalfa.

### Responses of *MsWOXs* to exogenous hormones

Phytohormones are the main factors for plant growth and development, which appear to have a strong connection to WOX transcription factors [30, 39, 56]. However, the direct linkage between *MsWOXs* and phytohormones in alfalfa has not been well established. In this study, the promoter regions 3.0 kb upstream of the translation start site of *MsWOXs* were analyzed, and we found a variety of phytohormone-responsive cis-elements including auxin, gibberellic acid (GA), and abscisic acid (ABA) (Fig. 4), and we determined the effects of exogenous phytohormone treatment on *MsWOX* gene expression (Fig. 5). It’s not surprising to see that most *MsWOX* transcripts were changed significantly under phytohormone treatments, given that both phytohormones and WOX genes are important developmental regulators. *MsWUS* was induced obviously in the shoots after cytokinin 6-BA treatment, which is consistent with the report that Arabidopsis WUS positively regulates cytokinin signaling by directly repressing type *A-ARRs* which negatively regulate the CK signal pathway [57]. WOX9 has been demonstrated as an effector of CK signaling in Arabidopsis and CK degradation in Medicago and tobacco [29, 39], and here we found that *MsWOX9* was induced obviously under 6-BA treatment (Fig. 5C, I), suggesting that MsWOX9 function in cytokinin homeostasis is conserved in alfalfa. In other species, cucumber *CsWOX3* and *CsWOX9* were upregulated by ABA and IAA, while *Dendrobium centum DCaWOX3(a/b)*, and *DCaWOX13(a/b)* negatively responded to ABA but positively responded to IAA [43, 58], and in Brachypodium, BdWOX12, 14 and 15 were shown to be activated by exogenous 6-BA, NAA, and GA, respectively [50]. These findings together indicate that *WOX* genes regulate various developmental pathways in close association with phytohormones, but the underlying molecular mechanism of the linkage between WOX and phytohormone responses needs to be further studied.

### Responses of *MsWOXs* to different external environments

Plants can adapt to adverse situations by adjusting their metabolism and altering their morphology. Previous reports mainly focused on WOX regulation of plant development and growth, but few studies investigated the roles of WOXs in diverse stresses. Tomato *SlWUS* and Arabidopsis *AtWOX6* are involved in cold stress through CBF-independent pathways [42, 59]. Rice *OsWOX11* and *OsWOX13* have been reported to be involved in drought resistance [36, 44]. In the present study, numerous cis-elements related to abiotic stress response were observed in almost all *MsWOX* promoters (Fig. 4) and the subsequent expression tests indicated that *MsWOXs* participated in abiotic stresses too. The strong responses of *MsWOX9-1*, *13 − 1*, and *10*/*14* to PEG treatment, and *MsWOX3*, *9 − 1*, and *10*/*14* to cold stress, are consistent with the analysis of cis-regulators in the promoters (Figs. 4, 6E, F and I-L and 7D-F, K and L). *MsWUS* and *MsWOX11* failed to respond to the PEG and low temperature, which might be due to the lack of corresponding cis-acting elements in their promoters (Figs. 4B, 6A and B and 7A and B).

Alfalfa is the most widely cultivated forage crop in the world, and forming a symbiosis with rhizobia for nitrogen-fixing nodules is a vital trait for the legume to improve yields. In this study, transcripts of *MsWOX3*, *9 − 1*, *9 − 2*, *13 − 1*, *13 − 2*, and *10*/*14* were highly expressed in nodules (Fig. 2), and their expression levels were measured after inoculation with *S.meliloti 1021*(Fig. 8). *MsWOX13-2* transcript was significantly accumulated continually (Fig. 8E), while *MsWOX3, 9 − 1*, and *10/14* were temporarily induced after 1 dpi (Fig. 8A, B, F), but *MsWOX9-2*, and *13 − 1* both showed no response to rhizobial inoculation (Fig. 8C, D). Previous research has reported that Medicago *MtWOX5* and pea *PsWOX5* were induced upon nodulation [60], which indicates that WOX family members participate in nitrogen-fixing processes. Since nodules as newborn organs form at the root cortex after infection by rhizobia, cell division, and differentiation are the most important steps during this biological process, which implies MsWOXs might be involved via hormone cross-talk. More detailed investigation of MsWOXs in the nodulation process is required including local expressions of certain *MsWOXs* using GUS staining or GFP fluorescence during nodule development in alfalfa. A comprehensive analysis of the interconnection between phytohormones, nodulation, and specific MsWOX gene expression will advance our understanding of the regulatory steps of nodulation and biological nitrogen fixation.

## Conclusion

In this study, we performed a genome-wide analysis of *MsWOX* genes, and a total of 14 MsWOXs were identified and classified into three subclades in alfalfa cv. Zhongmu No.1. Tissue expression of *MsWOXs* genes revealed their specific involvement in different organs and developmental programs. Combining the cis-acting element identification of promoters and expression analyses of *MsWOXs* under different treatments indicated that MsWOXs are involved in multiple biological processes during plant development including modulation and adaptation to adverse environmental conditions. Our results form the basis and provide insight into the diversity and functional significance of *MsWOX* genes in plant growth and abiotic stress responses in alfalfa.

### Electronic supplementary material

Below is the link to the electronic supplementary material.

Supplementary Material 1

Supplementary Material 2

## Data Availability

The phylogenetic trees were deposited in tree base (http://treebase.org) under the following URL: http://purl.org/phylo/treebase/phylows/study/TB2:S30292?x-access-code=90307dc81897f970003313d11d2fd0a6&format=html. All other data generated or analyzed during this study are included in this published article and its Additional files.

## Abbreviations

2,4-D: 2,4-Dichlorophenoxyacetic acid
6-BA: 6-Benzylaminopurine
ABA: Abscisic acid
At: *Arabidopsis thaliana*
BLAST: Basic Local Alignment Search Rool
Chr: Chromosome
cv: Cultivarietas
Da: Daltons
DCa: *Dendrobium catenatum*
DPI: Days Post Inoculation
GA: Gibberellin
HB: Homeobox
Mt: *Medicago truncatula*
Ms: *Medicago sativa*
Os: *Oryza sativa*
PEG: Polyethylene glycol
PI: Isoelectric Point
PSORT: Protein subcellular localization prediction tool
Bd: *Brachypodium distachyon*
WOX: WUSCHEL-related homeobox
