# Supplementary material for: Characterization and expression profiles of WUSCHEL-related homeobox (WOX) gene family in cultivated alfalfa (Medicago sativa L.)
Source: BMC Plant Biol. 2023 Oct 6;23:471. doi: 10.1186/s12870-023-04476-5 (PMC10557229; doi:10.1186/s12870-023-04476-5)
Supplement: Supplementary file 1 — Supplementary Material 1 [file 12870_2023_4476_MOESM1_ESM.docx]

**Supplemental Table1: Primers used in this paper**

| Primer name | Primer sequences (5’-3’) |
| --- | --- |
| MsWUS-attB1-F | ggggacaagtttgtacaaaaaagcaggcttcATGGAACAGCCTCAACAACAAC |
| MsWUS-attB2-R | ggggaccactttgtacaagaaagctgggtcTTAATTAGCATAATCTGGTGACCTACA |
| MsWUS-NS-attB2-R | ggggaccactttgtacaagaaagctgggtcATTAGCATAATCTGGTGACCTACA |
| MsWOX3-attB1-F | ggggacaagtttgtacaaaaaagcaggcttcATGTCTCCACCTGGTTCATC |
| MsWOX3-attB2-R | ggggaccactttgtacaagaaagctgggtcCTAGTCTTTGATTCTTGTTGTTGTG |
| MsWOX3-NS-attB2-R | ggggaccactttgtacaagaaagctgggtcGTCTTTGATTCTTGTTGTTGTG |
| MsWOX9-attB1-F | ggggacaagtttgtacaaaaaagcaggcttcATGGCTTCATCAAATAGACACTG |
| MsWOX9-attB2-R | ggggaccactttgtacaagaaagctgggtcTCACTCTCCAGCATTTTTTCC |
| MsWOX9-NS-attB2-R | ggggaccactttgtacaagaaagctgggtcCTCTCCAGCATTTTTTCC |
| MsWOX13-attB1-F | ggggacaagtttgtacaaaaaagcaggcttcATGGTGAACATGATGGA |
| MsWOX13-attB2-R | ggggaccactttgtacaagaaagctgggtcTCAACCTGCCAAATTGTAG |
| MsWOX13-NS-attB2-R | ggggaccactttgtacaagaaagctgggtcACCTGCCAAATTGTAG |
| MsActin-qF | CAAAAGATGGCAGATGCTGAGGAT |
| MsActin-qR | CATGACACCAGTATGACGAGGTCG |
| MsSTF-qF | TCTCATGCCAAGACCAGTCC |
| MsSTF-qR | GAACTCACCACAACTGCTGC |
| MsWUS-qF | TCCCATCATTCAAAGAGCACCA |
| MsWUS-qR | GAAGCAGAAGAAAGCCCAGC |
| MsWOX2-qF | GTTTCCACTGCATCCAACTGG |
| MsWOX2-qR | CCCAAATACTCCCTCCGTCC |
| MsWOX3-qF | GCAAACACATCAGAAGGGGc |
| MsWOX3-qR | AGAGTCTTGAGAGGTTTAGTGCA |
| MsWOX4-qF | GGAGGGACAAGGTGGAATCC |
| MsWOX4-qR | TCTGCTTTTGTCTCTCGCGT |
| MsWOX5-qF | TGGCAAGATAGAGAGCAAGAATGT |
| MsWOX5-qR | TGCATGCATACTCCACTTCTCT |
| MsWOX6-qF | GGCAACCACTTCAAACTGCA |
| MsWOX6-qR | GCTGAAGATGTTGTAGCTGGC |
| MsWOX9-1-qF | CCCTTCTTCCACCACCACAA |
| MsWOX9-1-qR | CCATGTGGTTGAGCAGGAAC |
| MsWOX9-2-qF | GCCTCTCCCGATGAAACTGT |
| MsWOX9-2-qF | GGGGTGTGGGTGGTTGTAAA |
| MsWOX11-qF | AGTGGTCAACAAGGCATGGA |
| MsWOX11-qR | TTGATGTCTAGTGGCCCTGC |
| MsWOX12-qF | GCTGGTGTTGGTCTTGGTCT |
| MsWOX12-qR | CCCCTTGGCATTTCTGTTGG |
| MsWOX13-1-qF | CTCCTACACCGGTTCAGCTC |
| MsWOX13-1-qR | GCACGCCTATTTTGGAACCA |
| MsWOX13-2-qF | AGGTCAAAGGGAAAGCAGCA |
| MsWOX13-2-qR | CCTCTAAGTACTGGCTGCACA |
| MsWOX14-qF | ACAACTTAACATCGGGGCGT |
| MsWOX14-qR | GTGTGAAGTCGGCTCCATCT |
