# Supplementary material for: Characterization and expression profiles of WUSCHEL-related homeobox (WOX) gene family in cultivated alfalfa (Medicago sativa L.)
Source: BMC Plant Biol. 2023 Oct 6;23:471. doi: 10.1186/s12870-023-04476-5 (PMC10557229; doi:10.1186/s12870-023-04476-5)
Supplement: Supplementary file 2 — Supplementary Material 2 [file 12870_2023_4476_MOESM2_ESM.pptx]

## Slide 1
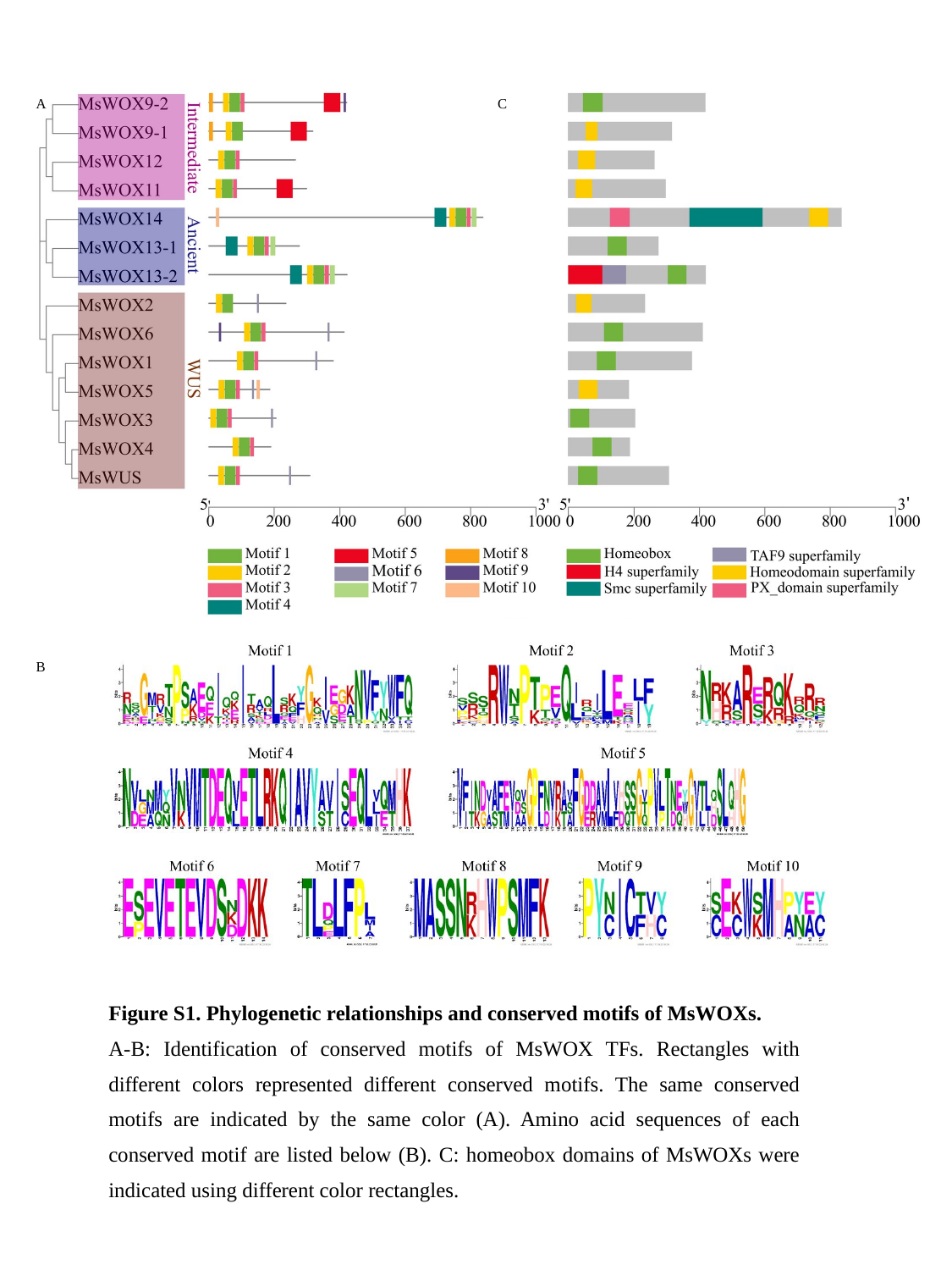

A
C
B
Figure S1. Phylogenetic relationships and conserved motifs of MsWOXs.
A-B: Identification of conserved motifs of MsWOX TFs. Rectangles with different colors represented different conserved motifs. The same conserved motifs are indicated by the same color (A). Amino acid sequences of each conserved motif are listed below (B). C: homeobox domains of MsWOXs were indicated using different color rectangles.

## Slide 2
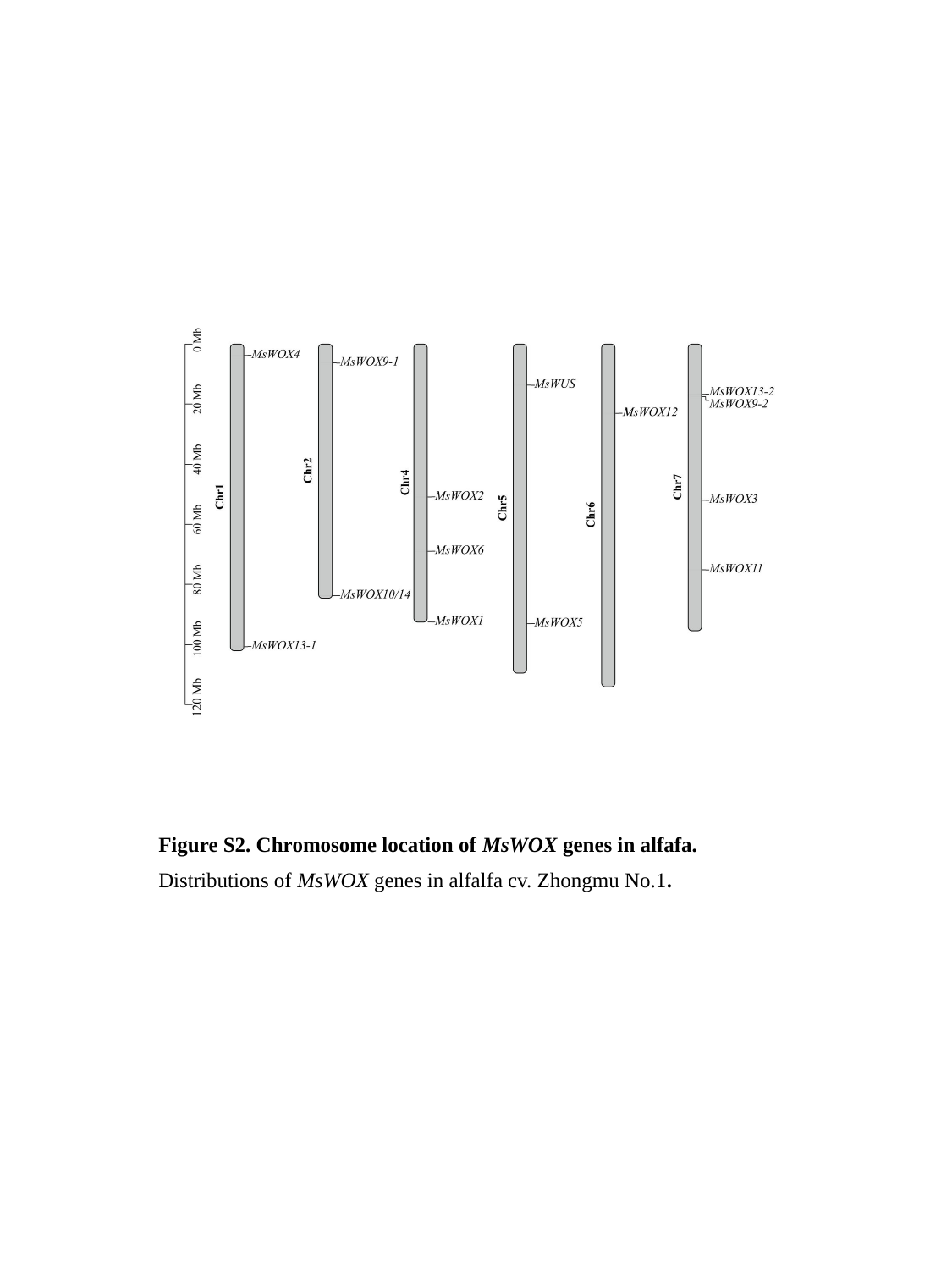

Figure S2. Chromosome location of MsWOX genes in alfafa.
Distributions of MsWOX genes in alfalfa cv. Zhongmu No.1.

## Slide 3
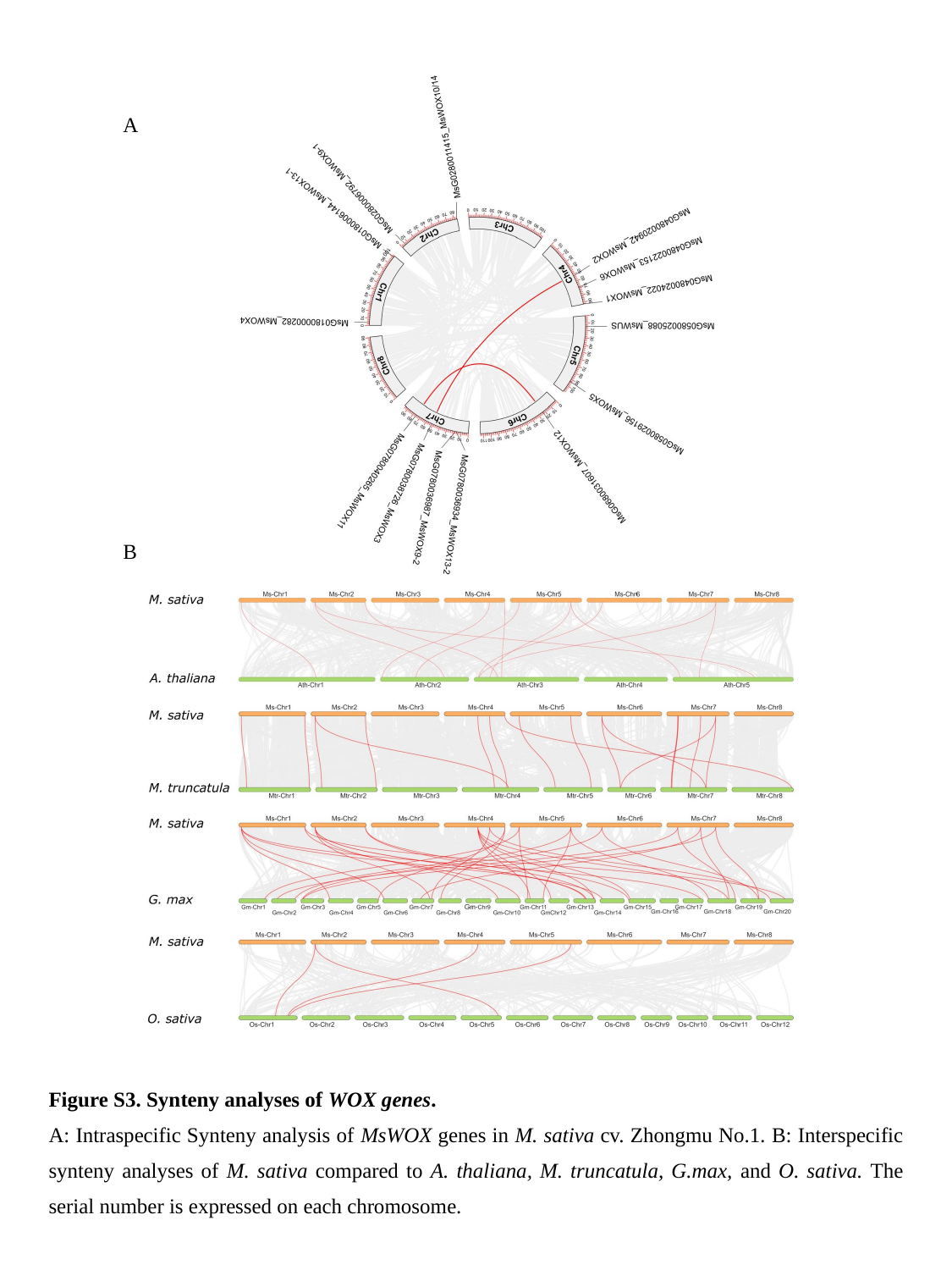

A
B
Figure S3. Synteny analyses of WOX genes.
A: Intraspecific Synteny analysis of MsWOX genes in M. sativa cv. Zhongmu No.1. B: Interspecific synteny analyses of M. sativa compared to A. thaliana, M. truncatula, G.max, and O. sativa. The serial number is expressed on each chromosome.
